# Factors Affecting Choice of Surgical Treatment of Cartilage Lesions of the Knee: An Analysis of Data From 5143 Patients From the German Cartilage Registry (KnorpelRegister DGOU)

**DOI:** 10.1177/23259671241255672

**Published:** 2024-07-25

**Authors:** Sebastian Gebhardt, Marcus Vollmer, Alexander Zimmerer, Ingo Rochel, Peter Balcarek, Philipp Niemeyer, Georgi I. Wassilew

**Affiliations:** † Center for Orthopaedics, Trauma Surgery and Rehabilitation Medicine, University Medicine Greifswald, Greifswald, Germany; ‡ Institute of Bioinformatics, University Medicine Greifswald, Greifswald, Germany; § Orthopädische Klinik Paulinenhilfe, Diakonie-Klinikum Stuttgart, Stuttgart, Germany; ‖ Klinik für Unfallchirurgie, Handchirurgie und Orthopädie, KRH Klinikum Nordstadt, Hannover, Germany; ¶ ARCUS Sportklinik, Pforzheim, Germany; # Department of Trauma Surgery, Orthopaedics and Plastic Surgery, University of Göttingen, Göttingen, Germany; ** OCM-Orthopädische Chirurgie München, München, Germany; †† Klinik für Orthopädie und Traumatologie, Universitätsklinikum Freiburg, Freiburg, Germany; Investigation performed at the Center for Orthopaedics, Trauma Surgery and Rehabilitation Medicine, University Medicine Greifswald, Greifswald, Germany

**Keywords:** articular cartilage, clinical assessment/grading scales, Deutsches KnorpelRegister (DGOU), epidemiology, German Cartilage Registry, knee, statistics

## Abstract

**Background:**

Symptomatic full-thickness cartilage lesions of the knee joint are considered an indication for cartilage repair surgery. Patient- and lesion-specific factors like age, nutritional status, etiology of defect, or integrity of corresponding joint surface remain controversial in indicating cartilage repair surgery. Furthermore, the selection of the most suitable cartilage repair technique for a specific cartilage lesion remains debatable.

**Purpose:**

To evaluate indications and choice of treatment method for cartilage repair surgery, depending on patient- and lesion-specific data from the German Cartilage Registry.

**Study Design:**

Cross-sectional study; Level of evidence, 3.

**Methods:**

A total of 6305 consecutive patients who underwent cartilage repair surgery of the knee evaluated and 5143 complete datasets were included in the analysis (follow-up rate, 81.5%). Patient-specific (age, body mass index, smoking status, previous operations, clinical leg axis) and lesion-specific (size, grading, location, etiology) data were provided by the attending surgeon at the time of surgery. Appropriate statistical tests were used to compare data depending on type and normality of data. Multivariable logistic regressions were calculated to investigate independent factors for the choice of specific cartilage repair techniques.

**Results:**

The median size of treated cartilage lesions was 3.6 cm^2^, and most defects were of degenerative origin (54.8%). Of the registered patients, 39.2% were categorized as overweight and 19.6% as obese, while 23.3% were smokers. The most prevalently documented operative techniques were the autologous chondrocyte implantation (ACI) (52.4%), bone marrow stimulation (BMS) (17.3%), and BMS augmented with collagen scaffolds (9.3%). Independent factors that made the use of ACI more likely were bigger lesion size, previous surgery at the joint, and lesions located at the trochlea or the patella. On the contrary, BMS or augmented BMS were preferred in older patients, with damaged corresponding joint surface, and with more concomitant surgeries.

**Conclusion:**

Cartilage repair surgery was indicated irrespective of nutritional status, smoking status, or etiology of the treated lesion. ACI was the most prevalent technique and was preferred for younger patients and patellar lesions. While older patients with degenerative changes to the joint were not excluded from cartilage repair surgery, the use of ACI was restricted.

Focal cartilage damage to the knee joint leads to impaired joint function and reduction of quality of life, representing a risk factor for the development of knee joint arthrosis.^49,56^ In addition to conservative therapy measures, various joint-preserving surgical techniques exist.^49^

According to expert recommendations, symptomatic focal cartilage lesions grades 3 or 4 on the International Cartilage Repair Society (ICRS) Score are considered an indication for a cartilage repair surgery.^4^ Traumatic cartilage lesions, intact corresponding joint surface and menisci, and patient age under 50 years were regarded as prerequisites for cartilage repair surgery, while degenerative cartilage lesions, bipolar cartilage lesions, obesity, and nicotine abuse, among others, were regarded as contraindications.^5^ Recent publications leave open the extent to which this strict indication has been revised, but in the meantime the majority of treated cartilage defects are degenerative in origin.^41^ Further studies have also shown cartilage repair surgery has been shown to be beneficial for patients who are tobacco users,^8^ older,^17^ and have bipolar cartilage lesions^11^ despite these being considered contraindications

Lesion size is the main parameter used to determine the best technique for cartilage repair. A recently published overview article provides a treatment algorithm that differentiates between chondral and osteochondral lesions and favors bone marrow stimulation (BMS) and associated techniques for the treatment of smaller cartilage lesions, while autologous chondrocyte implantation (ACI) is recommended for larger lesions.^24^ The Tissue Regeneration Working Group of the German Society for Orthopaedic an Trauma Surgery (Deutsche Gesellschaft für Orthopädie und Unfallchirurgie [DGOU]) has published and updated distinct treatment recommendations on indications and therapy for focal cartilage lesions of the knee joint over the last 2 decades.^4,5,39,41^ According to these recommendations, the minimum size of a cartilage lesion to be considered for an ACI was reduced from 3 to 4 cm^2^ to 2.5 cm^2^, and finally to 2 cm^2^. Accordingly, the maximum lesion size for an indication of BMS was reduced to 2 cm^2^. Recently, matrix-induced chondrogenesis (Matrix-BMS) was included as an alternative option for the treatment of medium-sized cartilage defects, from 1 to 4.5 cm^2^. Minced cartilage implantation (MCI) was also added as a potential technique, but there is insufficient evidence of proven results.^38^

In a previous investigation published in 2016, data from the German Cartilage Registry have already been evaluated concerning indications for cartilage repair surgery.^41^ This analysis of 1065 datasets revealed that indications were given mainly for degenerative cartilage lesions. ACI was the most prevalent technique and was used to treat bigger lesions compared to other techniques. Thus, the authors concluded that national and international guidelines were followed by the performing surgeons.^41^ In the meantime, the German Cartilage Registry has grown significantly.

The aim of this study was to evaluate the current development of indications for cartilage repair surgery, analyze current state-of-the-art cartilage repair treatment, and identify factors influencing the choice of surgical cartilage repair technique as documented by the German Cartilage Registry.

## Methods

The present analysis was carried out by evaluation of data from the German Cartilage Registry (KnorpelRegister DGOU). Since its initiation by the Clinical Tissue Regeneration Working Group (Arbeitsgemeinschaft Klinische Geweberegeneration) of the German Society for Orthopaedics and Trauma Surgery (DGOU) in 2013, treatment patterns and outcomes of patients after surgical treatment of cartilage defects at assigned centers in Germany, Austria, and Switzerland have been observed longitudinally. To date, the number of sites contributing data to the registry has increased to 120. This registry is conducted in accordance with the Declaration of Helsinki and registered at German Clinical Trials (DRKS00005617). Over 100 local ethics committees have approved the implementation of the registry in their sphere of influence to date. The current study was approved by the Ethics Commission of the University of Freiburg, Germany (EK-FR 105/13130795). The German Cartilage Registry is supported by research grants from Deutsche Arthrosehilfe e.V. and Oscar-Helene-Stiftung e.V. Mandatory requirements for participation of patients are legal age of majority; surgical treatment of cartilage defects of the knee, ankle, or hip joint at a participating site; signed written informed consent; and access to a private email address.

Since the registry began in 2013, data of 6305 patients who had undergone cartilage repair surgery of the knee were filed with the registry. The closing date of the evaluation was June 30, 2021.

A web-based RDE System (RDE-Light), developed by the Clinical Trials Unit (Freiburg) as an electronic data entry interface and data management system for clinical studies and other projects in clinical research, was used for data collection. With the described system data can be collected directly by an internet browser. The system is based on HTML- and PDF-format. RDE-Light is available in various languages, validated according to GAMP 5, and fulfils all requirements of good clinical practice. Furthermore, cryptographic security protocols (SSL/TLS), user authentication protocols, and authorization concepts are applied as a security standard.

Following informed consent, patients were registered to the database. At the time of surgery, the treating surgeon entered patient-, defect- and treatment-specific data into the registry. Patient-specific data entered included age at time of surgery, sex, body mass index (BMI), smoking status, number and type of previous operations at the specific joint or at the specific cartilage lesion, and clinical impression of the leg axis (neutral, varus, or valgus) according to a clinical investigation in standing or supine position. Lesion-specific data (size [in cm^2^], grading of the lesion [according to the ICRS], and etiology of the lesion [traumatic, posttraumatic, or degenerative]) were evaluated by the treating surgeon. In addition, the status of the menisci (intact, less than one-third resected, greater than one-third resected) was evaluated intraoperatively. Patients were contacted automatically via email, initially to assess immediate preoperative scores and second at 6, 12, 24, 60, and 120 months postsurgery for follow-up investigations. Subjective knee function was assessed using standardized instruments. In addition, patient satisfaction, complications, and revision surgeries were determined with self-administered tools. However, only baseline data collected at the time of surgery were analyzed for this study.

### Statistical Analysis

Registry data were screened for incorrect and implausible entries, which were filtered to reduce the bias in the subsequent analysis. Reasons for exclusion were missing size of the treated cartilage defect or the surgical method (n = 637); missing ICRS grading of the treated defect (n = 31); and missing or obviously incorrect entries concerning patient weight, height, BMI, and smoking status (n = 54). Because we suspected that the defect sizes were entered incorrectly, we considered only those cases with a minimum defect size of 25 mm^2^, resulting in the exclusion of further 122 datasets for subsequent analysis. Entries of other therapies were screened manually by 1 author (S.G.) and assigned to existing categories if possible. Patients without therapy or expressing doubts about therapy measures as suggested by free text entries were removed from the analysis set (n = 173). Further, 145 patients were excluded for receiving multiple or therapies other than ACI, ACI with bone graft, BMS, Matrix-BMS, osteochondral autograft transplantation (OCT), thermochondroplasty (TCP), or debridement. Free text entries of previous operations were reviewed manually by 1 author (S.G.) and recategorized as necessary. Thus, 5143 of 6305 datasets were considered complete and could be analyzed for the present analysis.

Data screening and statistical analysis were conducted with R Version 4.2.0 (R Core Team; https://www.R-project.org/). Summary measures include means and standard deviations or median with quartiles for continuous variables and counts for categorical or logical variables with row or column percentages. Summary tables were stratified by cartilage repair therapy, defect size, age quartiles, defect localization, and sex to allow univariable comparisons. Appropriate statistical tests were conducted depending on the variable type: Kruskal-Wallis rank sum test for non-normally distributed continuous variables, 1-way analysis of variance (ANOVA) to compare means of normally distributed variables, and Fisher’s exact test with simulated *P* values for count data. For a reasonably valid longitudinal analysis of the surgical techniques used, the proportions of each surgery year were calculated only from patients of those study centers that had continuously entered data into the German Cartilage Registry between 2015 and 2020. Two multivariable logistic regressions were calculated to investigate independent factors for the choice of ACI versus recommended BMS in defect sizes ≤2 cm^2^ and for ACI versus Matrix-BMS in defect sizes >2 cm^2^ but ≤4.5 cm^2^. We used an ACI-based stepwise procedure for variable selection in a complete case analysis. Age initially entered the regression function with natural cubic splines. After investigation of odds ratios (ORs) of age pairs, the regression formula was changed for better interpretability of the age effect. Based on the insignificance of age OR in patients younger than 45 years and quasi-linearly increasing OR thereafter, we decided to include age as a continuous variable with the number of years over the age of 45 years (setting the age for all patients ≤45 years to zero). Collinearity of independent factors was checked using variance inflation factors. ORs were computed with 95% confidence intervals (CIs) for each regression coefficient (for the increase of 1 unit in continuous variables or for each categorical level vs the reference level). Type 3 likelihood ratio tests were used to test each predictor variable in general for statistically significant effects on the therapy choice. An alpha-level of 5% was used.

## Results

Of the 6305 primarily created datasets, 5143 (81.6%) were included in the subsequent analysis. Reasons for exclusion were incompleteness or obvious implausibility of data entries. Overall, 113 different institutions contributed datasets to the analysis set. The 20 most active institutions accounted for 71.6% (n = 3681) of the registered cases. The single center that contributed the most cases was a privately run clinic specializing in orthopaedic sports medicine (n = 699). Among the 20 most active institutions were 3 university clinics, 2 state-run hospitals, and 15 privately run orthopaedic practices or clinics with a focus on orthopaedic sports medicine. A total of 43 institutions, including university clinics, state-run hospitals, and private practices, reported fewer than 10 cases in total.

The majority of treated patients were men (60.1%, n = 3089). The median age of the treated patients at time of surgery was 37 years (interquartile range [IQR], 27-47 years). The average BMI was 26.6 kg/m^2^± SD 4.8 kg/m^2^, with 39.2% of patients being categorized as overweight (BMI, 25.00-29.99 kg/m^2^; n = 2018) and 19.6% as obese (BMI ≥30 kg/m^2^; n = 1007). One quarter of the respondents (23.3%; n = 1196) were current smokers, while 4.8% (n = 247) were former smokers. The median size of treated cartilage defects was 3.6 cm^2^ (IQR, 2.0-5.0 cm^2^). The majority of treated cartilage defects were of degenerative origin 54.8% (n = 2815), compared with 21.4% (n = 1102) being of traumatic and 15.4% (n = 794) of posttraumatic origin. Treated cartilage defects were described predominantly as grade 3 (n = 1946; 37.8%) or 4 (n = 3115; 60.6%) according to ICRS.

In 60.5% (n = 3035) of cases, the corresponding joint surface was described as intact, according to intraoperative observation, while grade 1 or 2 degeneration was observed in 32.7% (n = 1643) and higher graded cartilage damage was reported in 6.8% (n = 339) of cases, respectively, according to ICRS. The majority of patients (n = 2767, 64.8%) had intact menisci at the time of cartilage repair surgery, while 21.6% (n = 923) were reported with a loss of up to one-third of the meniscal substance and an additional 9.2% (n = 392) of patients with a loss of more than one-third of the meniscal surface.

The anatomical axis was clinically examined in 4823 patients, with a neutral leg axis observed preoperatively in 69.5% (n = 3352) of patients. The impression of a clinical varus deformity was observed in 21.2% (n = 1021) of patients, while 9.3% (n = 450) of patients were evaluated with a valgus deformity. In 51.5% (n = 2651) of patients, the cartilage surgery was accompanied by a concomitant surgery. Most prevalent was a concomitant stabilization of the patella (n = 619, 12.0%), followed by osteotomies (n = 512, 10.0%), meniscal therapies (n = 447, 8.7%), and stabilization of cruciate or collateral ligaments (n = 223, 4.3%).

In 32.0% (n = 1645) of cases 1 previous surgery at the affected knee joint had been documented, 2 had been documented in 11.4% (n = 588), ≥3 in 6.7% (n = 347). These previous surgeries reported in 5136 patients had most often been meniscal therapies (n = 888, 17.3%), previous cartilage surgeries (n = 849, 16.5%), ligament surgeries (n = 497, 9.7%), or stabilization of the patella (n = 234, 4.6%). One previous cartilage surgery at the specific lesion had been documented in 16.7% (n = 855) of cases, and 3.4% (n = 177) of patients had already had ≥2 previous cartilage surgeries.

### Technique Used

**Figure 1.**
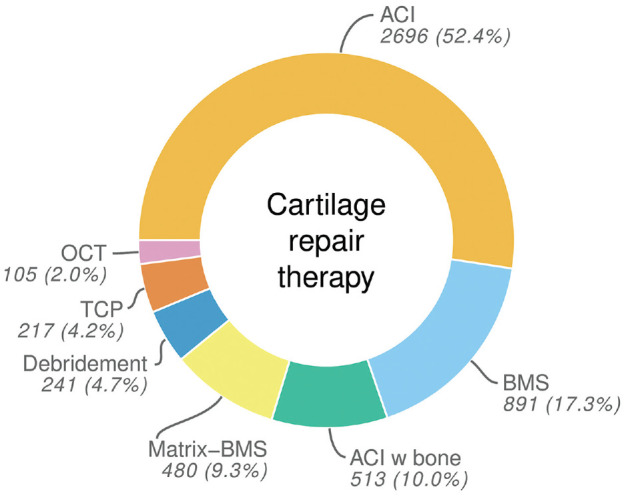
Overall distribution of different cartilage repair methods in the German Cartilage Registry. ACI, autologous chondrocyte implantation; BMS, bone marrow stimulation; Matrix-BMS, matrix-induced chondrogenesis; OCT, osteochondral autograft transplantation; TCP, thermochondroplasty.

The operative technique stated most often was ACI, with further cases being listed as ACI with additional bone graft augmentation. The second and third biggest groups of patients were treated by BMS and Matrix-BMS. Further techniques classified were Debridement, TCP, and OCT (Figure 1 and Table 1).

**Table 1:** Comparison of Patient- and Lesion-Specific Data Depending on Cartilage Repair Technique^*a*^

Variable	Overall N = 5143	Cartilage Therapy							*P* ^*b*^
		OCT N = 105 (2%)	BMS N = 891 (17.3%)	Debridement N = 241 (4.7%)	Matrix-BMS N = 480 (9.3%)	TCP N = 217 (4.2%)	ACI N = 2696 (52.4%)	ACI with Bone Graft N = 513 (10%)	
Age at surgery, y (median [IQR])	37 [27, 47]	36 [26, 47]	44 [31, 51]	48 [33, 56]	40 [28, 50]	50 [42, 54]	34 [27, 44]	28 [21, 38]	<.001
BMI, kg/m^2^, (mean [SD])	26.6 [4.8]	26.2 [3.6]	27.1 [5.0]	27.7 [4.8]	26.9 [5.1]	29.3 [7.3]	26.3 [4.4]	25.5 [4.3]	<.001
Lesion size, cm^2^ (median [IQR])	3.60 [2.00, 5.00]	1.00 [1.00, 1.54]	1.50 [1.00, 2.78]	2.25 [1.08, 4.00]	2.39 [1.50, 4.00]	3.00 [2.00, 5.00]	4.00 [3.00, 5.00]	4.00 [2.88, 5.60]	<.001
Sex, n (%)									.001
Male	3089 (60.1)	72 (68.6)	557 (62.5)	133 (55.2)	267 (55.6)	111 (51.2)	1623 (60.2)	326 (63.5)	
Female	2054 (39.9)	33 (31.4)	334 (37.5)	108 (44.8)	213 (44.4)	106 (48.8)	1073 (39.8)	187 (36.5)	
Etiology of defect, n (%)									<.001
Traumatic	1102 (21.4)	17 (16.2)	168 (18.9)	47 (19.6)	114 (23.8)	26 (12.0)	674 (25.0)	56 (10.9)	
Degenerative	2815 (54.8)	60 (57.1)	605 (67.9)	161 (67.1)	245 (51.0)	180 (82.9)	1366 (50.7)	198 (38.6)	
Posttraumatic	794 (15.4)	16 (15.2)	83 (9.3)	27 (11.2)	79 (16.5)	10 (4.6)	512 (19.0)	67 (13.1)	
Other	429 (8.3)	12 (11.4)	35 (3.9)	5 (2.1)	42 (8.8)	1 (0.5)	142 (5.3)	192 (37.4)	
Unknown	3	0	0	1	0	0	2	0	
Localization of defect, n (%)									<.001
Patella	1566 (30.5)	4 (3.8)	179 (20.1)	62 (25.7)	141 (29.4)	34 (15.7)	1065 (39.6)	81 (15.8)	
Trochlea	699 (13.6)	3 (2.9)	151 (17.0)	25 (10.4)	77 (16.1)	4 (1.8)	398 (14.8)	41 (8.0)	
Femoral condyle	2646 (51.5)	96 (91.4)	486 (54.7)	122 (50.6)	241 (50.3)	168 (77.4)	1157 (43.0)	376 (73.3)	
Tibial plateau	159 (3.1)	1 (1.0)	54 (6.1)	23 (9.5)	14 (2.9)	8 (3.7)	48 (1.8)	11 (2.1)	
Other	3 (0.1)	0 (0.0)	0 (0.0)	0 (0.0)	0 (0.0)	0 (0.0)	3 (0.1)	0 (0.0)	
More than 1 localization	63 (1.2)	1 (1.0)	19 (2.1)	9 (3.7)	6 (1.3)	3 (1.4)	21 (0.8)	4 (0.8)	
Unknown	7	0	2	0	1	0	4	0	
ICRS grading of treated defect, n (%)									<.001
1/2	82 (1.6)	1 (1.0)	14 (1.6)	55 (22.8)	1 (0.2)	1 (0.5)	8 (0.3)	2 (0.4)	
3a/b	1946 (37.8)	37 (35.2)	327 (36.7)	128 (53.1)	157 (32.7)	214 (98.6)	1028 (38.1)	55 (10.7)	
4a/b	3115 (60.6)	67 (63.8)	550 (61.7)	58 (24.1)	322 (67.1)	2 (0.9)	1660 (61.6)	456 (88.9)	
ICRS grading of corresponding joint surface, n (%)									<.001
Intact	3035 (60.5)	66 (62.9)	348 (39.5)	116 (49.8)	291 (64.8)	141 (67.5)	1722 (65.4)	351 (69.4)	
1/2	1643 (32.7)	34 (32.4)	381 (43.2)	78 (33.5)	129 (28.7)	63 (30.1)	810 (30.8)	148 (29.2)	
≥3	339 (6.8)	5 (4.8)	153 (17.3)	39 (16.7)	29 (6.5)	5 (2.4)	101 (3.8)	7 (1.4)	
Unknown	126	0	9	8	31	8	63	7	
Status of meniscus, n (%)									<.001
Intact	2767 (64.8)	59 (59.0)	403 (50.8)	84 (40.0)	253 (65.5)	31 (16.7)	1537 (72.5)	400 (84.4)	
<1/3 resected	923 (21.6)	23 (23.0)	203 (25.6)	46 (21.9)	81 (21.0)	101 (54.3)	415 (19.6)	54 (11.4)	
>1/3 resected	392 (9.2)	13 (13.0)	137 (17.3)	53 (25.2)	36 (9.3)	54 (29.0)	89 (4.2)	10 (2.1)	
Other	187 (4.4)	5 (5.0)	50 (6.3)	27 (12.9)	16 (4.1)	0 (0.0)	79 (3.7)	10 (2.1)	
Unknown	874	5	98	31	94	31	576	39	

a ACI, autologous chondrocyte implantation; ANOVA, analysis of variance; BMI, body mass index; BMS, bone marrow stimulation; ICRS, International Cartilage Repair Society; IQR, interquartile range; Matrix-BMS, matrix-induced chondrogenesis; OCT, osteochondral autograft transplantation; TCP, thermochondroplasty.

b Kruskal-Wallis rank sum test; 1-way ANOVA; Fisher’s exact test for count data with simulated *P* value (based on 2000 replicates).

**Figure 2.**
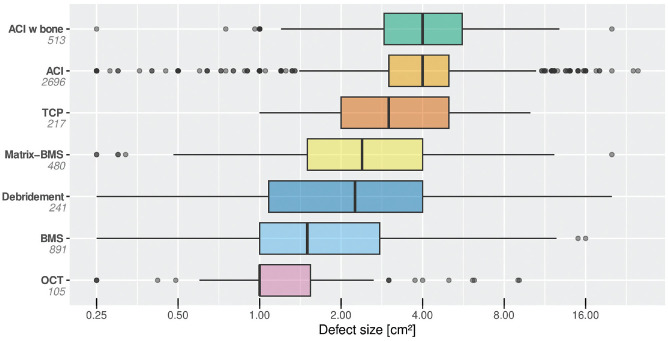
Comparison of median lesion size with IQR of the different cartilage repair techniques. ACI, autologous chondrocyte implantation; BMS, bone marrow stimulation; IQR, interquartile range; Matrix-BMS, matrix-induced chondrogenesis; OCT, osteochondral autograft transplantation; TCP, thermochondroplasty.

Differences between cartilage therapy methods were observed concerning average lesion size, average patient age, and BMI. The median age of patients undergoing ACI was younger and the mean BMI was lower compared with alternative methods. The median lesion size differed significantly between the techniques, with the biggest lesions size being observed for ACI and the smallest for OCT (Figure 2 and Table 1).

In comparison with other techniques, TCP was used mainly to treat cartilage lesions of degenerative origin. Approximately 60% of treatments with ACI, Matrix-BMS, BMS, and OCT were for cartilage defects grade 3 or 4 according to ICRS. TCP was used almost exclusively to treat grade 3 defects and ACI with bone grafting was used mainly in grade 4 defects. Deviating from this, debridement was used mainly to treat superficial cartilage defects (Table 1).

Although the corresponding joint surface was intact in the majority of treatments with ACI, Matrix-BMS, OCT, or TCP, the opposite was true for treatments with debridement and BMS (Table 1).

A partial meniscal resection of less than one-third of the meniscal surface was documented in one-fifth of patients in the ACI group, and more than one-third of the meniscus had been resected in 4.2% of cases. These proportions were significantly higher for other techniques (Table 1).

### Defect Size

According to the evidence-based treatment recommendations that were in place at the time of data sampling, treated defects were subdivided into 3 groups depending on lesion size. In the small defect group (<2.5 cm^2^), 1762 patients were registered compared with 1841 in the midsized defect group (2.5-4.0 cm^2^) and 1540 with big defects (>4.0 cm). While the average lesion size was significantly different between the groups, median age and average BMI were comparable (Table 2). Although statistically significant, there is no clinically meaningful difference in age.

**Table 2:** Comparison of Patient- and Lesion-Specific Data Depending on Defect Size^*a*^

Variable	Overall N = 5143	Defect Size			*P* ^*b*^
		<2.5 cm^2^ N = 1762 (34.3%)	2.5-4 cm^2^ N = 1841 (35.8%)	>4 cm^2^ N = 1540 (29.9%)	
Age at surgery, y (median [IQR])	37 [27, 47]	38 [27, 48]	36 [26, 46]	38 [28, 46]	<.001
BMI, kg/m^2^ (mean [SD])	26.6 [4.8]	26.5 [4.6]	26.6 [5.1]	26.8 [4.6]	.34
Lesion size, cm^2^ (median [IQR])	3.60 [2.00, 5.00]	1.50 [1.00, 2.00]	3.75 [3.00, 4.00]	6.00 [5.00, 7.00]	<.001
Sex, n (%)					.013
Male	3089 (60.1)	1017 (57.7)	1106 (60.1)	966 (62.7)	
Female	2054 (39.9)	745 (42.3)	735 (39.9)	574 (37.3)	
Cartilage repair therapy, n (%)					<.001
ACI	2696 (52.4)	456 (25.9)	1200 (65.2)	1040 (67.5)	
ACI with bone graft	513 (10.0)	111 (6.3)	180 (9.8)	222 (14.4)	
BMS	891 (17.3)	657 (37.3)	142 (7.7)	92 (6.0)	
Debridement	241 (4.7)	130 (7.4)	54 (2.9)	57 (3.7)	
Matrix-BMS	480 (9.3)	243 (13.8)	168 (9.1)	69 (4.5)	
OCT	105 (2.0)	94 (5.3)	6 (0.3)	5 (0.3)	
TCP	217 (4.2)	71 (4.0)	91 (4.9)	55 (3.6)	
Localization of defect, n (%)					<.001
Patella	1566 (30.5)	488 (27.7)	618 (33.7)	460 (29.9)	
Trochlea	699 (13.6)	272 (15.4)	220 (12.0)	207 (13.5)	
Femoral condyle	2646 (51.5)	885 (50.3)	932 (50.8)	829 (53.9)	
Tibial plateau	159 (3.1)	92 (5.2)	51 (2.8)	16 (1.0)	
Other	3 (0.1)	0 (0.0)	0 (0.0)	3 (0.2)	
More than 1 localization	63 (1.2)	24 (1.4)	15 (0.8)	24 (1.6)	
Unknown	7	1	5	1	
Concomitant operations, n (%)					<.001
No accompanying operations	2492 (48.5)	822 (46.7)	938 (51.0)	732 (47.5)	
Patellar stabilization	619 (12.0)	162 (9.2)	266 (14.4)	191 (12.4)	
Osteotomy	512 (10.0)	131 (7.4)	172 (9.3)	209 (13.6)	
Meniscal therapy	447 (8.7)	237 (13.5)	129 (7.0)	81 (5.3)	
Ligament stabilization	223 (4.3)	105 (6.0)	75 (4.1)	43 (2.8)	
Other	366 (7.1)	128 (7.3)	127 (6.9)	111 (7.2)	
Several accompanying operations	484 (9.4)	177 (10.0)	134 (7.3)	173 (11.2)	

a ACI, autologous chondrocyte implantation; ANOVA, analysis of variance; BMI, body mass index; BMS, bone marrow stimulation; IQR, interquartile range; Matrix-BMS, matrix-induced chondrogenesis; OCT, osteochondral autograft transplantation; TCP, thermochondroplasty.

b Kruskal-Wallis rank sum test; 1-way ANOVA; Fisher’s exact test for count data with simulated *P* value (based on 2000 replicates).

In the cohort with big cartilage defects, ACI was chosen in more than two-thirds of cases, followed by ACI with bone graft augmentation, BMS, Matrix-BMS, Debridement, TCP, and OCT. A similar distribution was observed for midsized defects. For the treatment of small cartilage defects, BMS was chosen more frequently than ACI and the proportion of treatments with Matrix-BMS was increased compared with big- and medium-sized defects (Table 2).

Although the rate of concomitant surgeries was comparable throughout all 3 groups, there are differences in the type of surgeries. For smaller defect sizes, more menisci were repaired; for larger defects sizes, more osteotomies and more patellar stabilizations and fewer ligament stabilizations were performed. The rate of 1 and 2 previous cartilage operations was higher in the large lesion-size group compared to the small- and midsized lesion groups (Table 2).

### Age

The investigated patient population was subdivided into 4 age quartiles, namely 10 to 27 years (n = 1373; median, 23 years; IQR, 19-25 years), 28 to 37 years (n = 1282; median, 32 years; IQR, 30-35 years), 38 to 47 years (n = 1277; median, 43 years; IQR, 40-45 years), and 48 to 78 years (n = 1211; median, 52 years; IQR, 50-56 years). With increasing age, the mean BMI increased and the largest median lesion size was observed in the age group 38 to 47 years.

**Figure 3.**
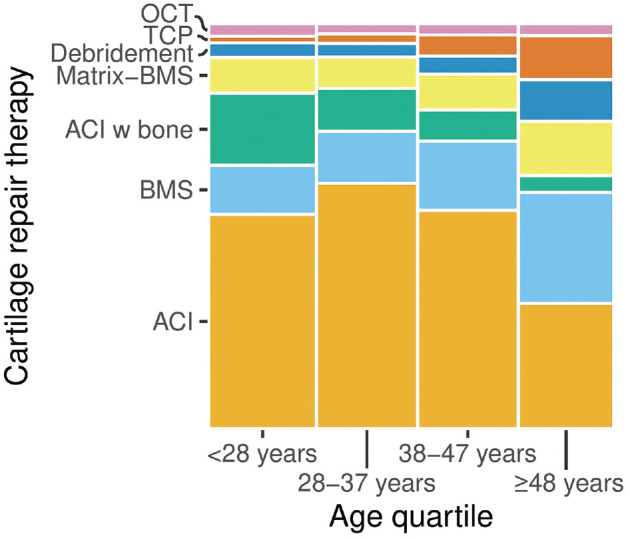
Distribution of different cartilage repair techniques depending on age quartiles. ACI, autologous chondrocyte implantation; BMS, bone marrow stimulation; Matrix-BMS, matrix-induced chondrogenesis; OCT, osteochondral autograft transplantation; TCP, thermochondroplasty.

ACI was used in the majority of cases for the first, second, and third age quartiles, with a significant decrease in the oldest quartile. Debridement was seldomly indicated for patients from the first to third quartile with a significant increase in the oldest quartile. Accordingly, the proportion of treatments with BMS, Matrix-BMS, and TCP was increased in the oldest quartile of patients (Figure 3 and Table 3).

**Table 3:** Comparison of Patient- and Lesion-Specific Data Depending on Groups Divided by Age Into Quartiles^*a*^

Variable	Overall N = 5143	Age Group				*P* ^*b*^
		<28 years N = 1373 (26.7%)	28-37 years N = 1282 (24.9%)	38-47 years N = 1277 (24.8%)	≥48 years N = 1211 (23.5%)	
Age at surgery, y (median [IQR])	37 [27, 47]	23 [19, 25]	32 [30, 35]	43 [40, 45]	52 [50, 56]	<.001
BMI, kg/m^2^ (mean [SD])	26.6 [4.8]	24.8 [4.4]	26.7 [4.3]	27.4 [5.0]	27.8 [5.0]	<.001
Lesion size, cm^2^ (median [IQR])	3.60 [2.00, 5.00]	3.00 [2.00, 4.40]	3.75 [2.25, 5.00]	4.00 [2.00, 5.00]	3.00 [1.50, 5.00]	<.001
Sex, n (%)						<.001
Male	3089 (60.1)	849 (61.8)	828 (64.6)	725 (56.8)	687 (56.7)	
Female	2054 (39.9)	524 (38.2)	454 (35.4)	552 (43.2)	524 (43.3)	
Cartilage repair therapy, n (%)						<.001
ACI	2696 (52.4)	764 (55.6)	819 (63.9)	724 (56.7)	389 (32.1)	
ACI with bone graft	513 (10.0)	248 (18.1)	132 (10.3)	92 (7.2)	41 (3.4)	
BMS	891 (17.3)	164 (11.9)	163 (12.7)	221 (17.3)	343 (28.3)	
Debridement	241 (4.7)	39 (2.8)	33 (2.6)	48 (3.8)	121 (10.0)	
Matrix-BMS	480 (9.3)	116 (8.4)	94 (7.3)	108 (8.5)	162 (13.4)	
OCT	105 (2.0)	32 (2.3)	22 (1.7)	25 (2.0)	26 (2.1)	
TCP	217 (4.2)	10 (0.7)	19 (1.5)	59 (4.6)	129 (10.7)	
Concomitant operations, n (%)						<.001
No accompanying operations	2492 (48.5)	633 (46.1)	648 (50.5)	662 (51.8)	549 (45.3)	
Patellar stabilization	619 (12.0)	297 (21.6)	191 (14.9)	99 (7.8)	32 (2.6)	
Osteotomy	512 (10.0)	66 (4.8)	112 (8.7)	158 (12.4)	176 (14.5)	
Meniscal therapy	447 (8.7)	47 (3.4)	46 (3.6)	105 (8.2)	249 (20.6)	
Ligament stabilization	223 (4.3)	66 (4.8)	71 (5.5)	55 (4.3)	31 (2.6)	
Other	366 (7.1)	111 (8.1)	90 (7.0)	90 (7.0)	75 (6.2)	
Several accompanying operations	484 (9.4)	153 (11.1)	124 (9.7)	108 (8.5)	99 (8.2)	
Localization of defect, n (%)						<.001
Patella	1566 (30.5)	605 (44.1)	475 (37.1)	301 (23.6)	185 (15.3)	
Trochlea	699 (13.6)	127 (9.3)	165 (12.9)	232 (18.2)	175 (14.5)	
Femoral condyle	2646 (51.5)	600 (43.8)	589 (46.0)	689 (54.0)	768 (63.5)	
Tibial plateau	159 (3.1)	27 (2.0)	38 (3.0)	32 (2.5)	62 (5.1)	
Other	3 (0.1)	0 (0.0)	3 (0.2)	0 (0.0)	0 (0.0)	
More than 1 localization	63 (1.2)	12 (0.9)	11 (0.9)	21 (1.6)	19 (1.6)	
Unknown	7	2	1	2	2	
Etiology of defect, n (%)						<.001
Traumatic	1102 (21.4)	459 (33.5)	314 (24.5)	186 (14.6)	143 (11.8)	
Degenerative	2815 (54.8)	437 (31.9)	605 (47.2)	842 (65.9)	931 (76.9)	
Post-traumatic	794 (15.4)	259 (18.9)	259 (20.2)	180 (14.1)	96 (7.9)	
Other	429 (8.3)	217 (15.8)	103 (8.0)	69 (5.4)	40 (3.3)	
Unknown	3	1	1	0	1	
Malformation clinical, n (%)						<.001
Neutral	3352 (69.5)	950 (74.5)	839 (70.0)	823 (68.4)	740 (64.6)	
Varus	1021 (21.2)	170 (13.3)	221 (18.4)	293 (24.4)	337 (29.4)	
Valgus	450 (9.3)	156 (12.2)	139 (11.6)	87 (7.2)	68 (5.9)	
Unknown	320	97	83	74	66	
Status of meniscus, n (%)						<.001
Intact	2767 (64.8)	869 (81.8)	756 (73.0)	664 (61.3)	478 (43.9)	
<1/3 resected	923 (21.6)	106 (10.0)	181 (17.5)	282 (26.0)	354 (32.5)	
>1/3 resected	392 (9.2)	39 (3.7)	60 (5.8)	93 (8.6)	200 (18.4)	
Other	187 (4.4)	48 (4.5)	38 (3.7)	44 (4.1)	57 (5.2)	
Unknown	874	311	247	194	122	
ICRS grading of corresponding joint surface, n (%)						<.001
Intact	3035 (60.5)	991 (73.9)	789 (62.9)	688 (55.7)	567 (47.8)	
1/2	1643 (32.7)	309 (23.0)	400 (31.9)	454 (36.7)	480 (40.5)	
≥3	339 (6.8)	41 (3.1)	66 (5.3)	94 (7.6)	138 (11.6)	
Unknown	126	32	27	41	26	

a ACI, autologous chondrocyte implantation; ANOVA, analysis of variance; BMI, body mass index; BMS, bone marrow stimulation; ICRS, International Cartilage Repair Society; IQR, interquartile range; Matrix-BMS, matrix-induced chondrogenesis; OCT, osteochondral autograft transplantation; TCP, thermochondroplasty.

b Kruskal-Wallis rank sum test; 1-way ANOVA; Fisher’s exact test for count data with simulated *P* value (based on 2000 replicates).

Concomitant surgeries were performed in approximately half of the cases throughout all age groups, with patellar stabilization surgeries being more prevalent in the youngest age group and osteotomies and meniscal therapies being more prevalent in the oldest age group.

Whereas lesions of the patella were most common in the youngest quartile cartilage, the femoral condyle was the region affected most frequently in patients of the oldest quartile. Furthermore, in the youngest age cohort, traumatic and degenerative cartilage lesions were distributed evenly around one-third of cases, while the majority of patients from the oldest quartile had degenerative defects.

Intact menisci were reported for 81.8% of patients in the youngest quartile and 73.0%, 61.3%, and 43.9%, respectively, for their older counterparts.

The corresponding joint surface was reported to be intact in 73.9% of patients of the youngest quartile, decreasing to 62.9%, 55.7%, and 47.8% for the second, third, and fourth quartile, respectively (Table 3).

### Location of Defect

The most common localization of treated cartilage defects was at the femur, followed by the patella, trochlea, and tibial plateau. The median age of the patients treated at the patella was lower compared with patients treated at the tibial plateau, femoral condyles, or the trochlea. Patients with cartilage damage of the patella were less likely to be overweight or obese compared with patients with cartilage damage of the femoral condyles, trochlea, or tibial plateau. The median defect size was similar for defects of the patella, the trochlea, and the femoral condyles, and smaller for tibial defects.

**Figure 4.**
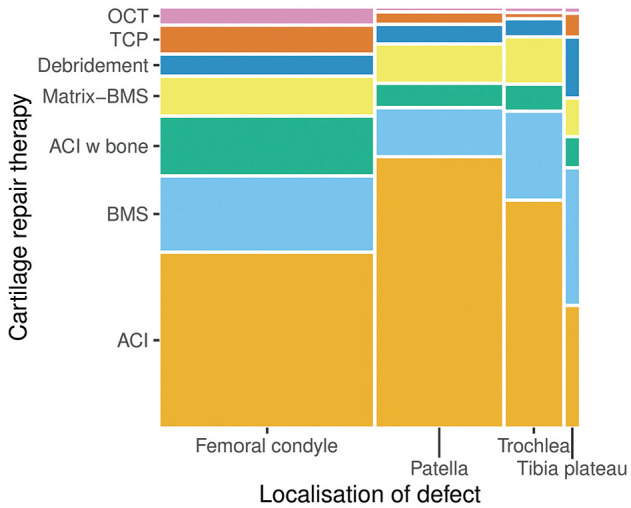
Distribution of different cartilage repair techniques depending on location of defect in the knee. ACI, autologous chondrocyte implantation; BMS, bone marrow stimulation; Matrix-BMS, matrix-induced chondrogenesis; OCT, osteochondral autograft transplantation; TCP, thermochondroplasty.

The therapies used frequently differed depending on the localization of the treated defect. At the patella, ACI was used in 68.0% of cases, followed by BMS (11.4%) and Matrix-BMS (9.0%). In comparison, at the femoral condyles, ACI was used in 43.7% of cases, BMS treatments in 18.4%, and Matrix-BMS was used in 9.1% of cases (Figure 4 and Table 4).

**Table 4:** Comparison of Patient- and Lesion-Specific Data Depending on Location of Treated Cartilage Defect in the Knee^*a*^

Variable	Overall N = 5070	Defect Localization				*P* ^*b*^
		Femoral Condyle N = 2646 (52.2%)	Patella N = 1566 (30.9%)	Trochlea N = 699 (13.8%)	Tibial Plateau N = 159 (3.1%)	
Age at surgery, y (median [IQR])	37 [27, 47]	40 [28, 49]	31 [24, 39]	40 [31, 48]	41 [30, 51]	<.001
BMI, kg/m^2^ (mean [SD])	26.6 [4.8]	26.9 [4.8]	26.0 [5.0]	26.8 [4.0]	26.8 [4.4]	<.001
BMI classification, n (%)						<.001
Underweight (<18.5 kg/m^2^)	57 (1.1)	26 (1.0)	22 (1.4)	9 (1.3)	0 (0.0)	
Normal (18.5-25 kg/m^2^)	2036 (40.2)	1001 (37.8)	750 (47.9)	224 (32.0)	61 (38.4)	
Overweight (25-30 kg/m^2^)	1987 (39.2)	1057 (39.9)	522 (33.3)	341 (48.8)	67 (42.1)	
Obese (>30 kg/m^2^)	990 (19.5)	562 (21.2)	272 (17.4)	125 (17.9)	31 (19.5)	
Lesion size, cm^2^ (median [IQR])	3.60 [2.00, 5.00]	3.75 [2.00, 5.00]	3.75 [2.25, 5.00]	3.36 [1.72, 5.00]	2.25 [1.50, 3.88]	<.001
Sex, n (%)						<.001
Male	3046 (60.1)	1659 (62.7)	750 (47.9)	533 (76.3)	104 (65.4)	
Female	2024 (39.9)	987 (37.3)	816 (52.1)	166 (23.7)	55 (34.6)	
Cartilage repair therapy, n (%)						<.001
ACI	2668 (52.6)	1157 (43.7)	1065 (68.0)	398 (56.9)	48 (30.2)	
ACI with bone graft	509 (10.0)	376 (14.2)	81 (5.2)	41 (5.9)	11 (6.9)	
BMS	870 (17.2)	486 (18.4)	179 (11.4)	151 (21.6)	54 (34.0)	
Debridement	232 (4.6)	122 (4.6)	62 (4.0)	25 (3.6)	23 (14.5)	
Matrix-BMS	473 (9.3)	241 (9.1)	141 (9.0)	77 (11.0)	14 (8.8)	
OCT	104 (2.1)	96 (3.6)	4 (0.3)	3 (0.4)	1 (0.6)	
TCP	214 (4.2)	168 (6.3)	34 (2.2)	4 (0.6)	8 (5.0)	
Concomitant operations, n (%)						<.001
No accompanying operations	2458 (48.5)	1329 (50.2)	628 (40.1)	430 (61.5)	71 (44.7)	
Patellar stabilization	612 (12.1)	39 (1.5)	529 (33.8)	44 (6.3)	0 (0.0)	
Osteotomy	502 (9.9)	409 (15.5)	30 (1.9)	39 (5.6)	24 (15.1)	
Meniscal therapy	439 (8.7)	310 (11.7)	40 (2.6)	55 (7.9)	34 (21.4)	
Ligament stabilization	223 (4.4)	169 (6.4)	21 (1.3)	27 (3.9)	6 (3.8)	
Other	361 (7.1)	140 (5.3)	144 (9.2)	69 (9.9)	8 (5.0)	
Several accompanying operations	475 (9.4)	250 (9.4)	174 (11.1)	35 (5.0)	16 (10.1)	
Malformation clinical, n (%)						<.001
Neutral	3315 (69.7)	1641 (65.4)	1097 (76.0)	479 (72.9)	98 (65.8)	
Varus	999 (21.0)	663 (26.4)	156 (10.8)	148 (22.5)	32 (21.5)	
Valgus	444 (9.3)	204 (8.1)	191 (13.2)	30 (4.6)	19 (12.8)	
Unknown	312	138	122	42	10	
Etiology of defect, n (%)						<.001
Traumatic	1092 (21.6)	534 (20.2)	409 (26.1)	119 (17.0)	30 (18.9)	
Degenerative	2767 (54.6)	1481 (56.0)	745 (47.6)	435 (62.2)	106 (66.7)	
Post-traumatic	783 (15.5)	353 (13.4)	306 (19.6)	110 (15.7)	14 (8.8)	
Other	425 (8.4)	276 (10.4)	105 (6.7)	35 (5.0)	9 (5.7)	
Unknown	3	2	1	0	0	
Status of meniscus, n (%)						<.001
Intact	2732 (65.0)	1441 (56.2)	850 (88.4)	362 (68.8)	79 (51.3)	
<1/3 resected	913 (21.7)	721 (28.1)	65 (6.8)	95 (18.1)	32 (20.8)	
>1/3 resected	376 (8.9)	290 (11.3)	10 (1.0)	41 (7.8)	35 (22.7)	
Other	184 (4.4)	111 (4.3)	37 (3.8)	28 (5.3)	8 (5.2)	
Unknown	865	83	604	173	5	
ICRS grading of corresponding joint surface, n (%)						<.001
Intact	3004 (60.7)	1545 (59.6)	1018 (66.8)	349 (51.6)	92 (59.4)	
1/2	1623 (32.8)	876 (33.8)	415 (27.2)	282 (41.7)	50 (32.3)	
≥3	321 (6.5)	170 (6.6)	92 (6.0)	46 (6.8)	13 (8.4)	
Unknown	122	55	41	22	4	

a ACI, autologous chondrocyte implantation; ANOVA, analysis of variance; BMI, body mass index; BMS, bone marrow stimulation; ICRS, International Cartilage Repair Society; IQR, interquartile range; Matrix-BMS, matrix-induced chondrogenesis; OCT, osteochondral autograft transplantation; TCP, thermochondroplasty.

b Kruskal-Wallis rank sum test; 1-way ANOVA; Fisher exact test for count data with simulated *P* value (based on 2000 replicates).

Concomitant surgeries were performed more frequently in cases of patellar cartilage defects compared with defects of the femur and trochlea. The most prevalent accompanying surgeries for cartilage lesions of the femur were osteotomies and meniscal therapies. In connection with cartilage lesions of the patella, stabilization surgeries of the patella were frequent.

A clinical impression of a leg axis deformity (valgus or varus) was more common in connection with cartilage lesions at the tibia and femur compared with cartilage lesions of the patella or trochlea. The proportion of traumatic cartilage injuries was highest for the patella and lowest for the trochlea. Accordingly, degenerative cartilage lesions were most prevalent at the tibial plateau, the trochlea, and the femoral condyles.

The meniscus was significantly more often partially resected with cartilage lesions at the femoral condyle, tibial plateau, and trochlea compared with the patella. However, the meniscal status was not reported in a significant proportion of treatments of the patella and the trochlea. The highest proportion of intact corresponding joint surface was reported for the patella and the lowest proportion for the trochlea (Table 4).

### Sex

A total of 60% of patients registered in the German Cartilage Registry were men. Although the mean age of male patients was younger, the mean cartilage lesion size was larger, and the BMI was comparable between the sexes. However, the proportion of male patients who were normal or underweight was significantly smaller compared with female patients. Accordingly, the proportion of overweight and obese male patients was higher compared with women.

Only minor sex-specific differences were observed for choice of therapy and the total number of concomitant surgeries was similar between the groups. Concerning specific procedures, concomitant osteotomies were more prevalent in male patients and concomitant patellar stabilization was observed more frequently in female patients.

It was documented that women were more often subject to revision cartilage repair surgery compared with men (22.4% vs 18.6%). Concerning further previous surgeries, a stabilization operation of the patella had been performed more often in women (6.9% vs 3.0%), and men had more frequently received a ligament stabilization surgery (12.1% vs 6.1%) or meniscal therapy (19.0% vs 14.7%).

The femoral condyles were the anatomic region treated most frequently in both sexes. Differences were observed in the frequency of cartilage repair treatments at the patella, which were more common in female patients. Trochlear defects were more frequently treated in male patients.

Whereas a clinically observed neutral leg axis was more prevalent in women, a varus deformity was observed more often in men and a valgus deformity was more prevalent in women.

The proportion of traumatic cartilage lesions was greater in men and the proportion of degenerative cartilage lesions was greater in women.

The corresponding joint surface and the menisci were slightly more often reported to be intact in women (Table 5).

**Table 5:** Comparison of Patient- and Lesion-Specific Data Depending on Sex^*a*^

Variable	Overall N = 5143	Sex		*P* ^*b*^
		Male N = 3089 (60.1%)	Female N = 2054 (39.9%)	
Age at surgery, y (median [IQR])	37 [27, 47]	36 [27, 46]	38 [27, 48]	<.001
BMI, kg/m^2^ (mean [SD])	26.6 [4.8]	26.9 [4.2]	26.2 [5.5]	<.001
Lesion size, cm^2^ (median [IQR])	3.60 [2.00, 5.00]	3.75 [2.00, 5.00]	3.10 [2.00, 5.00]	.024
BMI classification, n (%)				<.001
Underweight (<18.5 kg/m^2^)	58 (1.1)	21 (0.7)	37 (1.8)	
Normal (18.5-25 kg/m^2^)	2060 (40.1)	1052 (34.1)	1008 (49.1)	
Overweight (25-30 kg/m^2^)	2018 (39.2)	1443 (46.7)	575 (28.0)	
Obese (>30 kg/m^2^)	1007 (19.6)	573 (18.5)	434 (21.1)	
Cartilage repair therapy, n (%)				.002
ACI	2696 (52.4)	1623 (52.5)	1073 (52.2)	
ACI with bone graft	513 (10.0)	326 (10.6)	187 (9.1)	
BMS	891 (17.3)	557 (18.0)	334 (16.3)	
Debridement	241 (4.7)	133 (4.3)	108 (5.3)	
Matrix-BMS	480 (9.3)	267 (8.6)	213 (10.4)	
OCT	105 (2.0)	72 (2.3)	33 (1.6)	
TCP	217 (4.2)	111 (3.6)	106 (5.2)	
Concomitant operations, n (%)				<.001
No accompanying operations	2492 (48.5)	1481 (47.9)	1011 (49.2)	
Patellar stabilization	619 (12.0)	296 (9.6)	323 (15.7)	
Osteotomy	512 (10.0)	385 (12.5)	127 (6.2)	
Meniscal therapy	447 (8.7)	261 (8.4)	186 (9.1)	
Ligament stabilization	223 (4.3)	155 (5.0)	68 (3.3)	
Other	366 (7.1)	214 (6.9)	152 (7.4)	
Several accompanying operations	484 (9.4)	297 (9.6)	187 (9.1)	
Number of previous operations on the joint, n (%)				.066
0	2562 (49.8)	1516 (49.1)	1046 (50.9)	
1	1645 (32.0)	1016 (32.9)	629 (30.6)	
2	588 (11.4)	365 (11.8)	223 (10.9)	
≥3	347 (6.7)	192 (6.2)	155 (7.5)	
Unknown	1	0	1	
Number of previous operations on cartilage, n (%)				.002
0	4103 (79.9)	2511 (81.4)	1592 (77.6)	
1	855 (16.7)	484 (15.7)	371 (18.1)	
≥2	177 (3.4)	89 (2.9)	88 (4.3)	
Unknown	8	5	3	
Localization of defect, n (%)				<.001
Patella	1566 (30.5)	750 (24.3)	816 (39.8)	
Trochlea	699 (13.6)	533 (17.3)	166 (8.1)	
Femoral condyle	2646 (51.5)	1659 (53.8)	987 (48.1)	
Tibial plateau	159 (3.1)	104 (3.4)	55 (2.7)	
Other	3 (0.1)	2 (0.1)	1 (0.0)	
More than 1 localization	63 (1.2)	38 (1.2)	25 (1.2)	
Unknown	7	3	4	
Malformation clinical, n (%)				<.001
Neutral	3352 (69.5)	1925 (66.6)	1427 (73.9)	
Varus	1021 (21.2)	804 (27.8)	217 (11.2)	
Valgus	450 (9.3)	163 (5.6)	287 (14.9)	
Unknown	320	197	123	
Etiology of defect, n (%)				<.001
Traumatic	1102 (21.4)	738 (23.9)	364 (17.7)	
Degenerative	2815 (54.8)	1611 (52.2)	1204 (58.6)	
Posttraumatic	794 (15.4)	490 (15.9)	304 (14.8)	
Other	429 (8.3)	248 (8.0)	181 (8.8)	
Unknown	3	2	1	
ICRS grading of corresponding joint surface, n (%)				.041
Intact	3035 (60.5)	1782 (59.1)	1253 (62.6)	
1/2	1643 (32.7)	1023 (33.9)	620 (31.0)	
≥3	339 (6.8)	212 (7.0)	127 (6.3)	
Unknown	126	72	54	
Status of meniscus, n (%)				<.001
Intact	2767 (64.8)	1654 (62.9)	1113 (67.9)	
<1/3 resected	923 (21.6)	578 (22.0)	345 (21.0)	
>1/3 resected	392 (9.2)	264 (10.0)	128 (7.8)	
Other	187 (4.4)	133 (5.1)	54 (3.3)	
Unknown	874	460	414	

a ACI, autologous chondrocyte implantation; ANOVA, analysis of variance; BMI, body mass index; BMS, bone marrow stimulation; ICRS, International Cartilage Repair Society; IQR, interquartile range; Matrix-BMS, matrix-induced chondrogenesis; OCT, osteochondral autograft transplantation; TCP, thermochondroplasty.

b Kruskal-Wallis rank sum test; 1-way ANOVA; Fisher’s exact test for count data with simulated *P* value (based on 2000 replicates).

### Logistic Regression

Logistic regression was calculated to investigate for independent variables influencing decision-making of surgeons treating focal cartilage defects of the knee joint. Two situations were compared: factors influencing the choice between ACI and BMS for cartilage defects measuring <2.0 cm^2^ and factors influencing the decision between ACI and Matrix-BMS for cartilage lesions measuring between 2.0 and 4.5 cm^2^.

**Figure 5.**
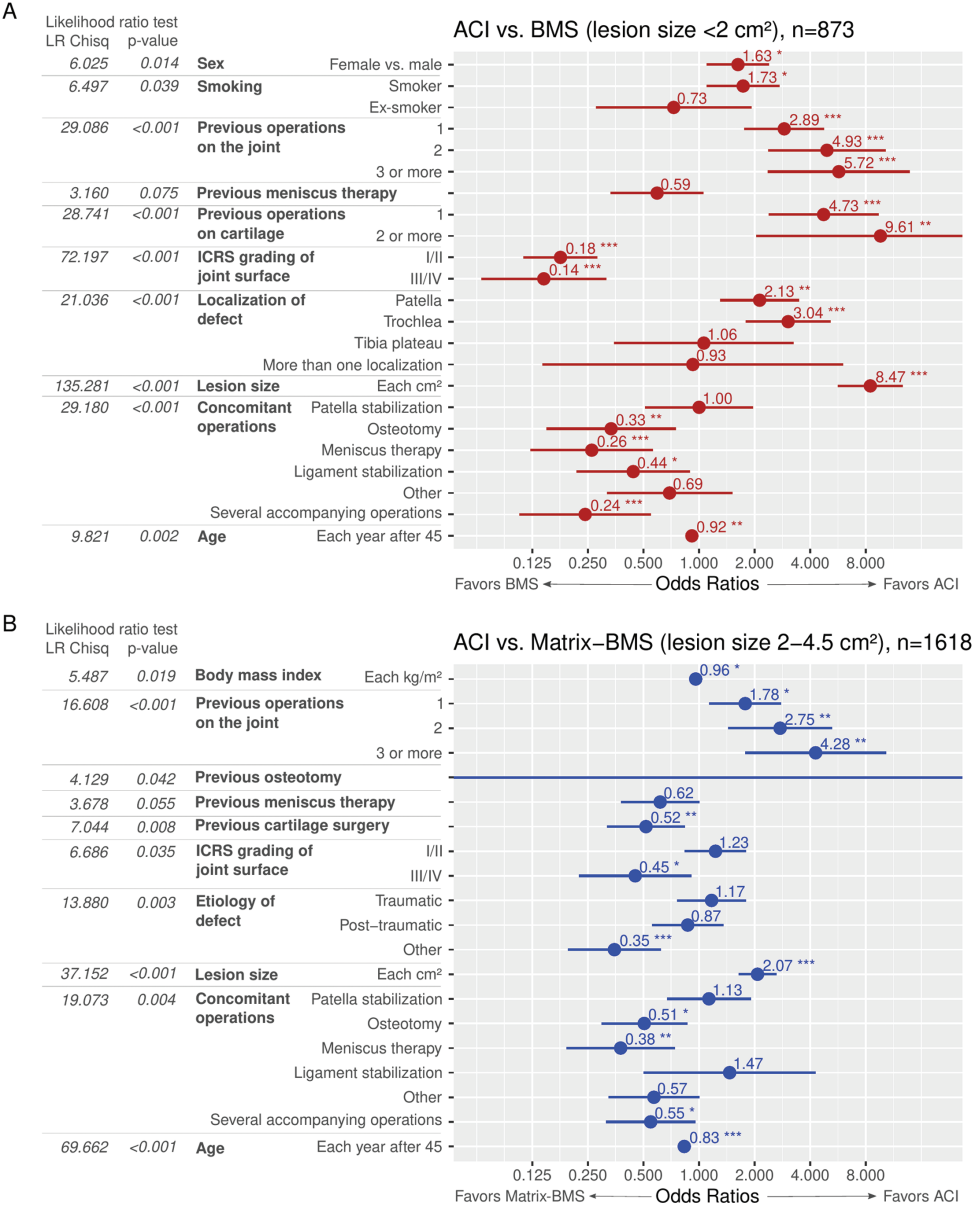
Impact of independent factors from multivariable logistic regression influencing decision-making for the treatment of cartilage lesions measuring (A) <2 cm^2^ and (B) between 2.0 and 4.5 cm^2^. ORs were computed for ACI surgery versus BMS or Matrix-BMS, respectively. (*P < .05, **P < .01, ***P < .001). ACI, autologous chondrocyte implantation; BMS, bone marrow stimulation; matrix-BMS, matrix-induced chondrogenesis; logistic regression chi-square test, logistic regression analysis of variance chi-square test; OR, odds ratio.

In the first logistic regression, a total of 873 cases (295 ACI and 578 BMS) were included to evaluate for independent factors influencing the choice of technique to treat focal cartilage lesions <2.0 cm^2^. The analysis revealed that in cases where the lesion size exceeded the specified limit (≥3, ≥2, or 1 previous surgery at the joint), surgeons preferred using ACI to treat lesions located at the trochlea or the patella, particularly in smoking patients and female patients. BMS was preferred in cases with damaged corresponding joint surface, accompanying meniscal therapies, accompanying osteotomies, and accompanying ligament stabilization surgeries (Figure 5A).

In the second logistic regression, a total of 1618 (1410 ACI and 208 Matrix-BMS) were included to evaluate for independent factors influencing the choice of technique to treat focal cartilage lesions measuring between 2.0 and 4.5 cm^2^. The analysis revealed that, in cases with bigger lesions within the given limit and more previous surgeries at the joint, ACI was preferred. Matrix-BMS was preferred in cases with damaged corresponding cartilage ≥grade 3, concomitant meniscal therapies, and concomitant osteotomies (Figure 5B).

### Longitudinal Observations

**Figure 6.**
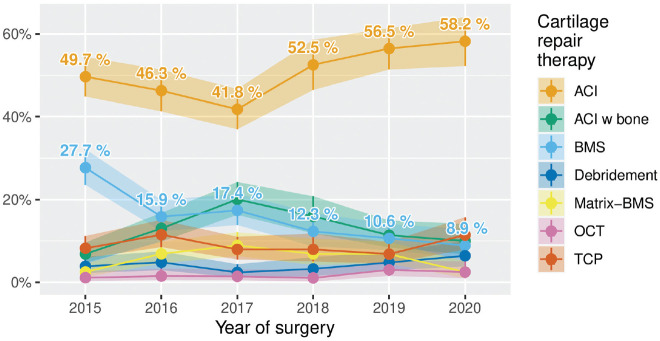
Proportion of different used techniques in longitudinal observation between 2015 and 2020, only including data from centers that contributed regularly over the investigated time period. ACI, autologous chondrocyte implantation; BMS, bone marrow stimulation; Matrix-BMS, matrix-induced chondrogenesis; OCT, osteochondral autograft transplantation; TCP, thermochondroplasty.

Longitudinal data could be collected from 12 contributing centers that added datasets on a regular basis between 2015 and 2020 into the cartilage registry. Thus, a subanalysis of 2193 datasets was performed. Over the course of 6 years, the proportion of ACI increased from 49.7% to 58.2%, while the proportion of BMS decreased from 27.7% to 8.9%. Further techniques remained at a similar level throughout the investigated period (Figure 6).

## Discussion

The main findings of the present analysis of data from the German Cartilage Registry were that obesity, smoking, and degenerative cartilage lesions were not considered exclusions for the indication for cartilage repair surgery. ACI was the most prevalent technique registered in the German Cartilage Registry and was used especially to treat lesions at the patella and trochlea. Older patients were treated less frequently with ACI compared with younger patients. Independent factors that affected the surgeon’s decision to use ACI to treat small cartilage defects were previous operations on the concerned joint or specific cartilage region, cartilage lesions that were located at the trochlea or at the patella, as well as patients that were female or smoked. BMS was favored in cases with patients >45 years old, damaged corresponding joint surface, and in connection with concomitant surgeries. For midsized cartilage defects, ACI was more likely to be chosen in cases with larger cartilage lesions and previous surgeries at the concerned joint, while Matrix-BMS was preferred in cases with patients >45 years old, damaged corresponding cartilage, and concomitant meniscal therapies or osteotomies.

### Indications and Contraindications for Cartilage Repair Surgery

The present analysis of data from the German Cartilage Registry revealed that the average patient indicated for cartilage repair surgery had a degenerative cartilage lesion, was 37 years old, was slightly overweight, and had a cartilage defect measuring 3.6 cm^2^ that was most likely located at the femoral condyles. While these patient-specific factors are comparable with other studies on cartilage repair surgery and are in line with everyday practice,^20,33^ they partly contradict early treatment recommendations that excluded degenerative cartilage lesions from cartilage repair surgery.^5^ However, recent research has revealed encouraging results concerning the surgical treatment of degenerative cartilage lesions and, to some extent, pushes the boundaries toward treatment of early osteoarthritis.^1,9,36^ According to the literature and the present analysis, the etiology of cartilage defects plays a minor role in decision-making for or against cartilage repair surgery, albeit the presented data do not allow any conclusions to be drawn about the outcome of surgical cartilage therapy for degenerative cartilage lesions in comparison with traumatic lesions, since no outcome data were analyzed.

Obesity is associated with the development of knee osteoarthritis and is associated with worse outcomes after cartilage repair surgery.^2,16,19,50,51^ Nevertheless, as documented by the German Cartilage Registry, the proportion of treated overweight patients (BMI between 25 and 30 kg/m^2^) was high at 39.2%, with an additional 19.6% of patients being obese (BMI >30 kg/m^2^). The proportions of overweight and obese patients in the registry mirror the proportions in the general population in Germany.^61^ It can be concluded that nutritional status does not play a role in decision-making for or against cartilage repair surgery, notwithstanding the above mentioned unfavorable evidence.

Preclinical studies revealed a dose-dependent negative influence of nicotine on chondrogenesis in vitro.^12^ A clinical investigation also showed a negative effect of smoking on the outcome after ACI.^25^ However, analysis of data from the German Cartilage Registry to investigate for the influence of smoking on outcomes after ACI found no statistically significant differences between smokers, former smokers, and nonsmokers.^8^ In the present analysis, 23.4% of treated patients admitted to smoking, which is a higher proportion than in the general German population.^43^ Thus, it can be concluded that, in current practice, smoking status does not play a role in decision-making for or against a cartilage repair surgery.

With emerging experience in cartilage repair surgery, the exclusion of bipolar cartilage lesions from cartilage repair is discussed. A previous review article revealed that minor lesions of the corresponding joint surface are increasingly tolerated, whereas corresponding full-thickness cartilage lesions remain a reason for exclusion.^29^ Successful cartilage repair of bipolar lesions with ACI in mid- to long-term follow-up has been documented.^44,45^ In an earlier analysis of data from the German Cartilage Registry, significant improvement from baseline data was found for uni- as well as bipolar cartilage lesions. Furthermore, differences in clinical outcome between the groups did not reach the minimal clinically important difference threshold. Thus, the authors concluded that patients with bipolar lesions should not be excluded from cartilage repair surgery.^11^ In the present analysis, partial-thickness cartilage lesions of the corresponding joint surface were tolerated in 32.7% and full-thickness cartilage lesions in 6.8% of cases. In conclusion, corresponding partial-thickness cartilage lesions are generally accepted, while full-thickness cartilage lesions might still be a restraint for cartilage repair surgery.

### Choice of Treatment Method

ACI is the surgical cartilage repair technique reported most prevalently in the German Cartilage Registry.^41^ The present analysis also showed that the portion of ACI compared with other documented cartilage repair techniques has further increased in recent years (Figure 6). The high proportion of ACI treatments found in this study could be biased since the German Cartilage Registry is not mandatory for all cartilage repair surgeries performed in Germany, and the high proportion of cases included by specialized centers might contribute to a higher proportion of ACI treatments than in general practice. The median defect size for the ACI was significantly higher, and median patient age and mean BMI were lower compared with other techniques. The increasing proportion of ACI treatments is understandable, as the ACI is the best studied cartilage repair technique and was shown to be superior to BMS in randomized controlled trials.^10,53,54^ Long-term efficacy of ACI has been proven,^48^ which, alongside the treatment recommendations that were in place at the time of data sampling,^39^ can serve as an explanation for the younger mean age of patients in the ACI group.

Superior outcomes of Matrix-BMS compared with BMS and ACI were found in a recently published meta-analysis.^27,33^ Furthermore, a randomized controlled trial found no difference between Matrix-BMS and ACI.^20^ However, the longitudinal observation over time (Figure 6) in the present study did not show an increase in the proportion of Matrix-BMS treatments in the German Cartilage Registry. Since Matrix-BMS was suggested as alternative treatment to ACI for the treatment of mid-sized cartilage lesions,^40^ the present analysis was extended by a logistic regression analysis to identify independent factors influencing treatment choice toward ACI or Matrix-BMS. The analysis revealed that higher patient age, full-thickness corresponding cartilage lesions, and concomitant osteotomies or meniscal therapies made the use of Matrix-BMS more likely in this group. On the other hand, ACI was preferred in younger patients, and those with previous joint operations and larger lesion size. It can be concluded that, despite increasing evidence of its value, Matrix-BMS is not regarded as equivalent alternative to ACI for the treatment of mid-sized cartilage defects but is preferred for the treatment of older patients and degenerative joints.

The decrease of treatments with BMS was observed not only in comparison with earlier analysis but also in the longitudinal observation of the present study (Figure 6).^41^ However, the BMS treatment group is still the second biggest in the German Cartilage Registry. The median age of treated patients and the mean BMI were higher compared with ACI and Matrix-BMS. A bigger lesion size was shown to negatively influence clinical outcome of BMS,^22,35^ and, in consequence, the indication for BMS according to treatment recommendations has been reduced from lesions of up to 4.0 to lesions of 2.0 cm^2^ or less,^38,41^ a trend that is partly mirrored by a relatively small median lesions size of 1.5 cm^2^ found in the present analysis. On the other hand, a high deviation with regard to defect size was observed for BMS (Figure 2), indicating that the BMS is used regularly to treat bigger cartilage lesions. It can be assumed that, in these cases, BMS is regarded as salvage procedure, as it is technically not demanding and is inexpensive.

The proportions of debridement and TCP were above average for treatments of degenerative cartilage lesions in the presence of partially resected menisci and damaged corresponding cartilage surface. It can be concluded from these observations that the technically less demanding and less expensive techniques are preferred in a degenerative environment.

Evidence on TCP is scarce as only 1 study group has focused on this technique and reported superior outcomes of TCP compared with debridement in the treatment of patients with grade 3 defects with concomitant meniscal lesions at 2-, 4- and 10-year follow-up.^57,58,59^ However, the population studied was reduced significantly due to a high number of revision surgeries, conversions to knee arthroplasties, and loss to follow-up, bringing into question the long-term efficacy of TCP as well as cartilage debridement techniques at 10-year follow-up.

A relatively high proportion of debridements were registered for cartilage lesions grade 1 or 2 according to the ICRS grading system. This has to be taken into consideration when evaluating results of an earlier study with data from the German Cartilage Registry, in which the authors found the functional outcome had improved significantly 2 years after debridement; however, no significant pain relief was observed.^63^ With both debridement and TCP, cartilage is not restored but the cartilage surface is smoothened mechanically to reduce shear forces of uneven cartilage tissue^21^; thus, the comparability of these techniques to attempt cartilage restoration that is made by the other techniques has to be questioned.

The OCT made up only 2% of cases in the German Cartilage Registry. The treated median lesions were the smallest observed for all treatment techniques. This observation is comprehensive, as inferior results for the OCT in the treatment of bigger cartilage lesions requiring the transplantation of >1 osteochondral cylinder were reported.^7^ This approach is in line with current treatment recommendations.^37,38^ The registered cases referred only to the transplantation of autologous osteochondral tissue. The osteochondral allograft transplantation is not considered in the German Cartilage Registry.

MCI is an alternative cartilage repair technique that has recently been increasingly performed. While encouraging clinical results have been reported,^30,52^ controlled studies are absent. In the time period analyzed, MCI treatments have not been reported in the German Cartilage Registry. A corresponding category has been introduced since and the evaluation of the efficacy of MCI can be expected in future analysis of the German Cartilage Registry.

### Location of Defect

More than half of the treatments documented in the German Cartilage Registry were at the femoral condyles and one-third at the patella. Compared with the respective average, BMS was used increasingly at the femur and the ACI at the patella. While several studies reported worse outcomes of cartilage repair surgery at the patella compared with the femur in general,^3,23,42,46,47^ a controlled study showed better results for ACI compared with BMS for the treatment of patellar cartilage lesions.^34^ This finding is mirrored by the preference to choose ACI for the treatment of patellar cartilage lesions in the present study. The success of patellofemoral treatment is associated with concomitant treatment of patellofemoral malalignment or instability.^32^ Accordingly, concomitant patellar stabilization surgeries were performed in more than one-third of documented cases at the patella in the present analysis.

Patients who were treated at the femur were older, had a higher BMI, and a higher proportion of degenerative cartilage lesions compared with patients who were treated at the patella. These factors could have contributed to the observed contribution of diverging treatment choices among the groups. A relatively high proportion of treatments for the trochlea with Matrix-BMS and for the tibia with debridement stand out.

### Size of Defect

Cartilage lesion size is regarded as a major variable to distinguish between different cartilage repair techniques according to current treatment recommendations.^24,38^ The national treatment recommendations that were in place at the time of data sampling for the present analysis suggested to use BMS for lesions measuring up to 3 to 4 cm^2^ and ACI for lesions measuring >3 to 4 cm^2^. For younger and physically more active patients, ACI was recommended starting from 2.5 cm^2^.^4,37^ The majority of patients with large lesions received an ACI treatment in line with the above-mentioned recommendations; however, one-fifth of the patients in this group received an alternative treatment technique. The BMS was the most prevalently used technique for small lesions; however, the proportion of ACI treatments made up for one-fourth of treatments in this group. This led to the consideration to investigate for independent variables influencing the decision-making in favor of ACI in small defects. A logistic regression calculation revealed that revision cartilage surgery, previous operations at the joint, bigger cartilage lesion size within the given limit of 2.5 cm^2^, female sex, and positive smoking status were predictors for the use of ACI in small cartilage lesions. On the contrary, BMS was preferred in cases with lesions of the corresponding joint surface and in the presence of concomitant surgeries. In absence of investigations comparing different cartilage repair techniques for revision cartilage repair surgery, the ACI was regarded as the best option according to the present analysis. Furthermore, the use of BMS instead of ACI is more likely in degenerative situations. The association between concomitant surgeries and BMS could be explained for practical reasons, as BMS treatments are technically less demanding and less time consuming. However, it has to be considered that good clinical results have been reported for concomitant treatment with anterior cruciate reconstruction and ACI.^31^ Thus, the effort of ACI treatment should be considered despite concomitant surgical procedures.

### Patient Age

Unlike earlier recommendations that excluded patients >50 years old from cartilage repair surgery,^5^ recent treatment recommendations do not set a certain age limit.^38^ The long-term efficacy of ACI in the treatment of cartilage defects of older patients has been reported.^17^ In light of demographic change and increased number of older patients with high functional demand, the debate is likely to continue, and the expansion to cartilage repair even in early stage osteoarthritis has been discussed.^9^ The population was divided into age quartiles to investigate the influence of age in the present analysis. In the oldest quartile, with a median age of 52 years, the proportion of ACI treatments was reduced significantly compared with the younger quartiles. On the other hand, treatments with Matrix-BMS, BMS, debridement, and TCP increased. Furthermore, the performed logistic regression analysis revealed that, with older age, the likelihood of indications for BMS and Matrix-BMS compared with ACI increased. Thus, it can be concluded that, whereas older patients are no longer excluded from cartilage repair surgery, the use of ACI is still restricted despite previously reported favorable results of ACI.^17^

### Sex

Women are more prone to develop knee osteoarthritis than men.^60^ Knee function in the presence of focal cartilage lesions has been reported to be lower in women compared with men.^55^ Furthermore, female sex negatively influenced the outcome of ACI treatment for cartilage lesions of the knee joint.^28^ Presumably, hormonal differences account for a reduced thickness and resistance of cartilage and lead to accelerated cartilage degeneration in women.^13,14,18,26,62^ It was therefore remarkable that, in the present analysis, more male than female patients with cartilage defects of the knee were treated. The proportion of overweight female patients was also significantly lower compared with male patients. Concerning defect location, women more frequently received treatments at the patella, while in men treatments at the femoral condyles and the trochlea were more likely. Although the proportion of treatments with ACI was distributed evenly, women were more likely to receive debridement and TCP, while men were treated more frequently with BMS. A previous analysis of data from the German Cartilage Registry confirmed worse overall functional outcome of cartilage repair surgery for women but revealed a higher relative increase of patient-reported scores in women after cartilage repair surgery.^15^ For the interpretation of worse results of cartilage repair surgery in women, the higher proportion of cartilage defects at the patella has to be taken into account as a confounding factor, as these defects are reported with worse outcomes.^3,42^

Whereas this study provides thorough insight into different patient-specific or cartilage lesion-specific factors contributing to decision-making in cartilage repair surgery, it does not provide outcomes data. Thus, it was not designed to conclude whether the presented decisions concerning indications for surgical cartilage repair in general or specific surgical cartilage repair techniques were appropriate with regard to clinical outcome or cost-effectiveness.

### Limitations

Besides several advantages, data taken from registries are associated with certain issues. The present analysis needs to consider the particularly frequent inconsistences in collected data and reporting, as well as the issue of selection bias, which are both problematic.^6^ Due to inconsistencies in data collection and implausible data entries, 1017 of the initial 6305 data entries had to be excluded from further analysis. In several cases, regrouping was necessary because they were initially classified under BMS but upon further examination it was found that collagen gel was used, which should have been indicated in a free-text field. Similarly, in some ACI cases listed as revision surgery, the only procedure performed previously was the mandatory cartilage cell harvesting. The stated lower limb alignment was not necessarily provided by an objective radiological assessment but was a clinical evaluation without specification of exact measured values. Data on secondary metabolic, cardiovascular, or hormonal diseases, as well as regularly used medication or dietary habits, are not considered as possible confounders by the registry. It can furthermore be assumed that the remarkably high proportion of ACI treatments recorded in the German Cartilage Registry is due to a selection and reporting bias, with the majority of cases coming from several high-volume centers, and might not represent general practice as entering data into the German Cartilage Registry is done on a voluntary basis and is not obligatory. For the future, a mandatory inclusion of all cartilage therapies in the registry would be desirable. However, the limitation of selection bias does not account for those parts of the present analysis that did not refer directly to the chosen treatment method, like the analysis concerning indications for cartilage repair surgery in general, the location of defect, size of defect, patient age, or sex. Concerning the chosen treatment method, the assumed influence of selection and reporting bias was addressed by evaluating the proportion of a reported treatment method under certain circumstances in comparison with the average reported proportion of that method.

## Conclusion

For patients registered in the German Cartilage Registry, cartilage repair surgery was indicated irrespective of patient age, nutritional status, or smoking status. The majority of treated cartilage defects were of degenerative origin, and the ACI was by far the most prevalently recorded treatment method. The ACI was also used in a significant number of cases to treat small cartilage lesions. Further independent factors making the decision for an ACI treatment more likely were younger patient age, relatively larger lesion size within the given limit, previous operations on the concerned joint or specific cartilage region, cartilage lesions that were located at the trochlea or at the patella, as well as in female patients or smoking patients. On the other hand, BMS and Matrix-BMS were more likely to be chosen for patients with damaged corresponding joint surface and with concomitant surgeries. The use of ACI in older patients with degenerative changes to the joint was restricted.
